# Editorial: On the Origin and Function of Human NK-Like CD8+ T Cells: Charting New Territories

**DOI:** 10.3389/fimmu.2017.01588

**Published:** 2017-11-15

**Authors:** Fernando A. Arosa

**Affiliations:** ^1^Faculty of Health Sciences, CICS-UBI, Health Sciences Research Center, University of Beira Interior, Covilhã, Portugal

**Keywords:** CD8+ T cells, suppressor, regulatory, aging, cancer, infection, inflammation, homeostasis

**Editorial on the Research Topic**

**On the Origin and Function of Human NK-Like CD8+ T Cells: Charting New Territories**

Human effector-memory CD8+ T cells displaying characteristics of NK/innate cells are a heterogeneous pool of multifunctional lymphocytes that are predominantly found in peripheral tissues and organs (1–3). In terms of phenotype, functional plasticity, and localization, NK-like CD8+ T cells are in a privileged place to regulate basic physiological processes, from immune protection against pathogens to wound healing and tissue regeneration, as shown for other T cell populations (4–6). The goal of this research topic was to bring novel insights into the origin and function of human NK-like CD8+ T cells.

The notion that NK-like CD8+ T cells share innate and adaptive features is elegantly described by Pereira and Akbar. In their review, they summarize the phenotypic, functional, and transcriptomic features that shape the generation of NK-like CD8+ T cells during human aging, including the signaling pathways involved. The authors propose that human NK-like CD8+ T cells are not dysfunctional, but a distinct T cell population that compensates for functional defects of conventional NK and CD8+ T cells. In line with this thinking, Michel et al. propose that the increase in NK-like CD8+ T cells seen in aged healthy people represents a remodeling of the T cell compartment to cope with physical and cognitive deleterious changes that take place with aging. In their review, they show an association between certain NK-like CD8+ T cell subsets and healthy aging, which led them to propose that there are subsets of NK-like CD8+ T cells that are bioindicators of successful aging and longevity. Although CMV seropositivity has long been considered a driving force behind the expansions of NK-like CD8+ T cells seen in the elderly, this view is changing. Thus, Saavedra et al. report data regarding the lack of association between CMV seropositivity and accumulation of NK-like CD8+ T cells in the elderly. By studying a cohort of CMV-infected young and elderly healthy Cubans, they found that age, but not CMV, was the main driving factor influencing the expansions of NK-like CD8+ T cells, which is in agreement with the studies referred by Michel et al. These data point to aging-related factors, among others, as responsible for the NK-like CD8+ T cell expansions, as discussed in the article of Pita-López et al. By performing a comprehensive analysis of the phenotypic characteristics, function, and development of human NK-like CD8+ T cells in the context of aging, autoimmunity, cancer, and infection, they conclude that the molecular cues responsible for the generation of NK-like CD8+ T cells as well as their exact function is still a matter of debate that warrants further investigations.

Although NK-like CD8+ T cells is a relatively recent designation, these cells were originally described as suppressor, as discussed in a more focused context by Xu et al. The authors engage in a comprehensive historical review of the phenotypic and functional features of human suppressor CD8+ T cells in the context of transplantation, autoimmune diseases, and viral infection, highlighting their CD28−, KIR+, CTLA-4+, PD-L1+, and Foxp3+ phenotype. They carefully review the mechanisms used by suppressor CD8+ T cells to inhibit T cell responses, highlighting the role of the inhibitory receptor ILT3 expressed by dendritic cells, and discuss novel immunosuppressive therapies. The origin and function of human NK-like CD8+ T cells were reviewed in a broader context by Arosa et al. In view of the expression of a highly diverse array of innate receptors, including receptors that trigger amphiregulin secretion such as IL-18R/IL-33R/ST2 (5), the authors envision NK-like CD8+ T cells as a highly experienced population with the skills and expertise to sense and cope with alterations that take place within an ever-changing environment, to keep tissues and organs intact and functional, in part by way of tissue regeneration. The authors also propose that open MHC class I conformers expressed by metabolically active cells, dividing cells, and stressed cells are important players that need to be taken into account to understand NK-like CD8+ T biology. In this regard, Cardoso and Arosa discuss the possible bone protective role of a subset of gingival NK-like CD8+ T cells during periodontitis. Based on recent data, the authors propose that gingival CD8+ T cells contain a pool of NK-like CD8+ T cells with regulatory/suppressor function, and these cells could be involved in the maintenance of alveolar bone integrity by constitutively downregulating inflammation under homeostatic conditions and initiating repairing mechanisms in case of tissue injury. The authors also acknowledge that under overt pathogenic bacterial colonization this protective role may be surpassed by the bacterial immune response and led to bone loss.

The surpassing of the proposed protective side of NK-like CD8+ T cells within inflamed tissues is addressed by Hodge and Hodge. The authors review recent data, including their own, pointing to populations of NK-like CD8+ T cells as involved in the inflammation of the airways in patients with chronic obstructive pulmonary disease. Importantly, the authors discuss recent data indicating that inflammatory NK-like CD8+ T cells are resistant to steroid treatment, reinforcing the need for novel therapeutic approaches. In line with this study, Lourenço et al. discuss the possible functional roles of NK-like CD8+ T cells in asthma. Based on the current knowledge, the authors propose a model where CD8+ T cells with the NK-like phenotype could exert pro-inflammatory, regulatory/suppressor or tissue regenerative activities, depending on the cytokine composition of the lung microenvironment. However, they also conclude that further phenotypical and functional studies are necessary for a better classification of these different subtypes of NK-like CD8+ T cells.

De Andrés et al. show original data suggesting that in pregnant women with multiple sclerosis (MS) there is activation of a population CD3+CD8+CD56+ T cells and at the same time a remission of disease activity, akin to an increase in sex hormones. Since this is not observed in non-pregnant women with MS, the data suggest that a subset of regulatory NK-like CD8+ T cells that are activated during pregnancy could play a role in ameliorating MS. However, the factors mediating this activation remain to be elucidated. One of the features of regulatory/suppressor CD8+ T cells is the production of the anti-inflammatory cytokines IL-10 and TGF-β. In this regard, Vuddamalay and van Meerwijk review data comparing classical CD8+CD28− Treg, which express KIR receptors and are therefore NK-like CD8+ T cells, with CD8+CD28low Treg. Although both populations produce IL-10 and TGF-β upon activation, CD8+CD28− Treg originate in the periphery while CD8+CD28low Treg are thought to originate in the thymus. The possible regulatory role of CD8+CD28low Treg in certain immunopathologies is discussed.

Finally, two related articles bring attention to the characterization of innate-like CD8+ T cells in humans and their relevance in cancer. In the first article, and based on their recent identification of a new subset of human NK-like CD8+ T cells expressing KIR, NKG2A, ST2, and Eomes, and rapidly producing IFN-γ upon IL-12/IL-18 triggering, Jacomet et al. provide experimental evidence that chronic myeloid leukemia (CML) is associated with quantitative and functional deficiencies of innate CD8+ T cells that were corrected upon CML remission, suggesting that innate CD8+ T cells may contribute to CML control. In the second paper, Barbarin et al. delineate the putative pathways and signals that could lead to the generation of innate CD8+ T cells capable of controlling cancer development, namely, the cytokines IL-4, IL-12, IL-15, and IL-18, iNKT cells, and the transcriptions factors PLZF and Eomes. In all, these two studies propose that in humans, innate CD8+ T cells constitute a new lymphocyte population that could have an important role in antitumor immunity.

In summary, the articles of this research topic provide novel insights into the mechanisms and conditions that drive the accumulation of NK-like CD8+ in humans and the possible roles played by these multifunctional lymphocytes. Considering their capability of sensing various environmental signals, studies addressing their functional response to physiological challenges, namely, endogenous products released by stressed, injured, or dead cells, will certainly further our understanding into these experienced human CD8+ T cells.

## Author Contributions

The author confirms being the sole contributor of this work and approved it for publication.

## Conflict of Interest Statement

The author declares that the research was conducted in the absence of any commercial or financial relationships that could be construed as a potential conflict of interest.
